# Development and internal validation of a risk prediction model for refractory hypoalbuminemia in peritoneal dialysis patients to guide nutritional intervention

**DOI:** 10.3389/fnut.2026.1950177

**Published:** 2026-10-29

**Authors:** Limin Zhang, Desen Duan, Suyu Lu, Min Wang, Yujie Wang, Huaying Pei

**Affiliations:** Department of Nephrology, The Second Hospital of Hebei Medical University, Hebei, China

**Keywords:** clinical prediction model, diabetes mellitus, hypoalbuminemia, malnutrition, nomogram, peritoneal dialysis, peritoneal protein excretion, refractory hypoalbuminemia

## Abstract

**Background:**

Hypoalbuminaemia is closely related to poor prognosis in patients with PD. In clinical practice, we found that hypoalbuminemia is difficult to correct and persists longer in some PD patients. The primary purpose of this study was to analyze the cause of refractory hypoalbuminemia in PD patients and construct a clinical prediction model to help identify these patients at an early stage.

**Methods:**

we retrospectively enrolled PD patients treated at the Second Hospital of Hebei Medical University between January 2021 and January 2023. Clinical data, laboratory results, peritoneal equilibration test (4hD/Pcr), and peritoneal protein excretion rate were collected. Patients were divided into modeling and internal validation cohorts. Risk factors were screened by LASSO regression and included in a multivariable logistic model to construct a nomogram. Model performance was evaluated by discrimination, calibration, and decision curve analysis (DCA).

**Results:**

a total of 368 PD patients were included, of whom 132 (35.9%) had refractory hypoalbuminemia. Independent risk factors included diabetes mellitus, elevated C-reactive protein (CRP), and high 4hD/Pcr; protective factors included higher BMI, triglycerides ≥1.265 mmol/L, HDL-C, and LDL-C. Peritoneal protein excretion rate was significantly higher in patients with refractory hypoalbuminemia and those with diabetes, and positively correlated with 4hD/Pcr (*r* = 0.6, *P* < 0.001). The prediction model showed excellent discrimination in the modeling cohort (AUC = 0.939) and internal validation cohort (AUC = 0.930), with good calibration and clinical net benefit. This study identifies key risk factors for refractory hypoalbuminemia in PD patients and presents a validated prediction model with high accuracy and clinical utility.

**Conclusion:**

this study identifies key risk factors for refractory hypoalbuminemia in PD patients and presents a internal validated prediction model with high accuracy and clinical utility, which can guide early intensive nutritional intervention.

## 1 Introduction

Peritoneal dialysis (PD), as one of the crucial modalities of renal replacement therapy, compared with hemodialysis, has advantages such as simple operation, home-based therapy (1), hemodynamic stability (2), and preservation of residual renal function (3), and has therefore been increasingly accepted and chosen by patients with end-stage renal disease worldwide (4).The continuous innovation of PD-related technologies has significantly reduced treatment-related complications. However, there are still some PD-related complications that affect the long-term survival of patients. A study that included 11,477 patients on peritoneal dialysis found that the 5-year survival rate for PD patients was only 54 percent (5). hypoalbuminemia is an independent risk factor for poor prognosis on peritoneal dialysis and is strongly associated with cardiovascular disease and dialysis failure; for every ten g/L drop in serum albumin level, PD patients have an approximately 4-fold increased risk of heart failure; the risk of death is increased by approximately 2-fold (6–8); At the same time, studies have shown that improving hypoalbuminemia in PD patients may increase their survival rate (9, 10). This suggests that we can significantly improve the prognosis of PD patients by actively correcting their hypoalbuminemia. Therefore, in clinical activities, healthcare professionals often improve the nutritional status of PD patients by giving oral α-keto acid and whey protein powder and educating patients to increase protein intake to improve their dietary structure. However, we still found that some PD patients with hypoalbuminemia had difficulty in correcting their serum albumin and remained in a long-term hypoproteinaemic state even after various interventions (medicine) were given. This phenomenon is called refractory hypoalbuminemia. Currently, the study of hypoalbuminemia is more limited to common hypoalbuminemia and less on this refractory hypoalbuminemia. At the same time, early clinical identification of PD patients with refractory hypoalbuminemia and timely intervention may reduce the incidence of refractory hypoalbuminemia and help to improve their prognosis. This study aims to construct a clinical prediction model by analyzing the factors affecting CAPD patients with refractory hypoalbuminemia to help the clinic quickly identify patients with high-risk refractory hypoalbuminemia, to reduce the incidence of refractory hypoalbuminemia and to improve the prognosis of patients.

## 2 Materials and methods

**Study Population:** previous studies have found that serum albumin reaches peak levels in PD patients on peritoneal dialysis for 1 year, irrespective of the initial albumin level, followed by a downward trajectory. Therefore, CAPD patients treated with PD for >1 year were included in this study. This was a single-center, retrospective study. The study population comprised patients receiving regular follow-up for continuous ambulatory peritoneal dialysis (CAPD) at the Peritoneal Dialysis Center of the Second Hospital of Hebei Medical University between January 1, 2021 and January 31, 2023,All patients received routine nutritional management and follow-up according to our center's standard practice. Inclusion criteria: (1) age≧18years; (2) patients who had been on CAPD for more than 1year. (3) complete clinical data.Exclusion criteria: (1) Previous history of hepatic decompensation and malignancy. (2) Follow-up interval greater than 3 months. (3) Acute infectious disease within 3 months. (4) significant volume overload. Volume overload was identified based on routine clinical assessment, including body weight changes, presence of peripheral or pulmonary edema, blood pressure, ultrafiltration volume, and history of heart failure. Patients judged to have significant volume overload by the treating clinician were excluded. This information was extracted retrospectively from the medical records.

The modeling cohort was enrolled from patients who received PD treatment at the peritoneal dialysis center of the Second Hospital of Hebei Medical University from January 1, 2021, to December 31, 2021. The temporal cutoff for the modeling cohort was December 31, 2021. Follow-up for all patients ended on January 31, 2023. Therefore, the available follow-up for patients in the modeling cohort was at least 13 months (from December 31, 2021 to January 31, 2023) and at most approximately 25 months (from January 1, 2021 to January 31, 2023). This follow-up duration was sufficient to assess refractory hypoalbuminemia, because its definition requires hypoalbuminemia to persist for more than 6 months.

**Data Collection:** we collected (1) demographic data, including Medical history, including cardiovascular history (e.g., coronary heart disease, heart failure, cerebrovascular disease, etc.), medication use, age, gender, BMI, length of dialysis (in months), results of Peritoneal equilibration test (PET) (4hD/Pcr was chosen as an indicator for evaluating peritoneal transport function), history of diabetes mellitus, history of medication administration of compound alpha-keto acids, and the presence of residual renal function(Residual renal function was defined as a 24-h urine volume ≥200ml/d. Patients with a 24-h urine volume < 200ml/d or anuria were considered to have no residual renal function. Data on 24-hour urine volume were extracted from the medical records). (2) Data from laboratory tests, including serum albumin, low-density lipoprotein cholesterol (LDL-C), high-density lipoprotein cholesterol (HDL-C), triglycerides(TG), urea clearance index Kt/v, C-reactive protein(CRP), glycosylated hemoglobin(HbA1c), and 24-h urine protein quantification. (3) The PD effluent of PD patients was collected, and the volume of effluent and its storage time were recorded. The concentration of micro-protein in the effluent was determined by the pyrogallol red method. The peritoneal protein excretion rate was calculated based on the formula: peritoneal protein excretion rate (mg/min) = (PD effluent protein concentration ^*^ effluent volume)/storage time of PD patients. All laboratory tests were performed by the Laboratory Department of the Second Hospital of Hebei Medical University; glucose peritoneal dialysis solution was used in all patients.

At the same time, we screened the best predictors of refractory hypoalbuminemia in PD patients, constructed a clinical prediction model based on the predictors' regression coefficients, plotted a nomogram to visualize the model, and internal validated the model.

Definition of refractory hypoalbuminemia: (1) serum albumin < 35 g/L; (2) duration of hypoalbuminemia >1 year; (3) hypoalbuminemia still exists after various interventions such as whey protein powder and educating patients to increase dietary protein intake. Patients who met all three criteria were called refractory hypoalbuminemia patients. All patients with hypoalbuminemia received routine nutritional interventions at our center. Group assignment was based on their status at the last follow-up. The patients were divided into a refractory hypoalbuminemia group and no refractory hypoalbuminemia group according to the presence or absence of refractory hypoalbuminemia.

## 2.1 Statistical analysis

(1) Quantitative information is expressed as mean ± standard deviation or median (interquartile spacing) after normality testing. Qualitative data were expressed as percentages. The Wilcoxon test and X^2^ test compared differences between groups.
(2) Influence factor analysis: the Box-Tidwell method was used to test for a linear relationship between the continuous independent variables and the logit transformed values of the dependent variable. The multicollinearity diagnosis was tested for the presence of multicollinearity between independent variables. Afterward, age, gender, BMI, length of dialysis (months), LDL cholesterol, HDL cholesterol, triglycerides, urea clearance index Kt/v, history of diabetes mellitus, history of compound α-keto acid medication, C-reactive protein, residual renal function, and 4hD/Pcr were included as independent variables in multifactorial logistic regression analysis.
(3) Propensity Score Weighting: diabetic patients were used as the case group, and non-diabetic patients were matched using propensity score weighting. Patients taking oral compounded alpha-keto acids were used as the case group, and propensity score weighting was used to match patients not taking oral compounded alpha-keto acids.
(4) To investigate the effect of mean glycated hemoglobin level on refractory hypoalbuminemia in patients with PD combined with diabetes mellitus by Wilcoxon test analysis.
(5) Predictive model construction: the best predictors of refractory hypoalbuminemia in PD patients were screened using Lasso regression analysis. Similarly, the Box-Tidwell method was used to test whether there was a linear relationship between the continuous independent variables and the logit transformed values of the dependent variable. After testing for multicollinearity between the best predictors, the predictors were included in a multifactor logistic regression model. A nomogram was constructed based on the regression coefficients.Internal validation was performed in the modeling cohort using both enhanced bootstrap and 10-fold stratified cross-validation. For the enhanced bootstrap, 1,000 resamples with replacement were drawn from the original modeling cohort. In each resample, the multivariable logistic regression model was refitted, and the AUC and Brier score were calculated on the original cohort. Optimism was defined as the apparent AUC minus the mean bootstrap AUC. The optimism-corrected AUC was calculated as the apparent AUC minus optimism. The same procedure was applied to the Brier score. For the 10-fold stratified cross-validation, patients were randomly divided into 10 folds, stratified by refractory hypoalbuminemia, so that each fold had a similar proportion of events. In each iteration, one fold served as the test set and the remaining nine folds as the training set. The model was refitted, and the AUC and Brier score were computed on the test set. This process was repeated 10 times, and the mean AUC and mean Brier score across the test folds were reported. Both bootstrap and 10-fold cross-validation are internal validation methods and cannot replace independent external validation. Because an external validation cohort that was independent at the patient level and had sufficient follow-up could not be constructed from the available dataset, no external validation was performed. Model discrimination was assessed in the modeling cohort using the area under the receiver operating characteristic (ROC) curve (AUC). Calibration was evaluated using calibration curves and Brier scores. Decision curve analysis was performed to evaluate the clinical utility of the model.
(6) The PD effluent of PD patients was collected, and the volume of effluent and the storage time were recorded. The concentration of micro-protein in the effluent was determined by the pyrocatechol red method. The peritoneal protein excretion rate was calculated according to the formula: peritoneal protein excretion rate (mg/min) = (protein concentration of PD effluent^*^volume of effluent)/time of storage to analyze the comparison of peritoneal protein excretion rate of patients with diabetes, the size of peritoneal protein excretion rate of patients with refractory hypoalbuminemia, and the correlation of peritoneal transport function and peritoneal protein excretion rate. We analyze and compare the size of the peritoneal protein excretion rate in patients with combined diabetes mellitus, the size of the peritoneal protein excretion rate in patients with refractory hypoalbuminemia, and the correlation between peritoneal transport function and peritoneal protein excretion rate.
(7) Comparison of the effect of glycated hemoglobin on refractory hypoalbuminemia in patients with diabetes mellitus combined with PD and the effect of 24-h annual protein quantification on refractory hypoalbuminemia in patients with residual renal function, analyzed by the Wilcoxon test. (Statistical analyses were performed via R 4.3.1)

## 3 Results

### 3.1 Baseline Characteristics

1.1 A total of 401 PD patients were screened; 10 patients had a previous history of hepatic decompensation or malignancy, 8 patients had an acute infectious disease within 3 months, and 15 patients had a follow-up interval of more than 3 months. Finally, 368 PD patients were included in this study. 25.8% of all patients had comorbid diabetes mellitus (95/368 patients); 18.5% were treated with compound alpha-keto acids (68/368 patients); 35.9% of the patients met the inclusion criteria for refractory hypoalbuminemia (132 /368 patients); and diabetes mellitus was more prevalent in the refractory hypoalbuminemia group (*p* < 0.05). Also, triglycerides, LDL cholesterol, BMI, and HDL cholesterol levels were lower in the refractory hypoalbuminemia group compared to patients without refractory hypoalbuminemia; C-reactive protein levels, 4hD/Pcr, were higher. (*p* < 0.05) (Table 1).

**Table 1:** Patient demographics, lab test results, past medical history, and medication usage.

Variable	No Refractory hypoalbuminemia *N* = 236	Refractory hypoalbuminemia *N* = 132	*p*-value
Age (years)	54 [45, 60]	56 [42, 63]	0.39
Females			0.93
Female	128 (54%)	71 (54%)	
Male	108 (46%)	61 (46%)	
BMI (kg/m2)	25.00 [19.50,26.70]	18.25 [17.15, 22.00]	**< 0.001**
Duration of PD (months)	60.00 [48.40,78.55]	55.60 [47.10,77.40]	0.39
**Compound** α**-Ketoacid Tablets**			0.47
No	195 (83%)	105 (80%)	
Yes	41 (17%)	27 (20%)	
**DM**			**< 0.001**
No	203 (86%)	70 (53%)	
Yes	33 (14%)	62 (47%)	
ALB (g/L)	38.65 ± 2.53	31.69 ± 2.51	**< 0.001**
TG (mmol/L)	1.72 [1.18,2.61]	1.12 [0.84,1.61]	**< 0.001**
HDL-C (mmol/L)	1.07 [0.87,1.21]	0.91 [0.77,1.07]	**< 0.001**
LDL-C (mmol/L)	2.58 [2.21,3.19]	2.09 [1.89, 2.34]	**< 0.001**
CRP (mg/L)	3.01 [1.87,4.97]	5.41 [2.86,10.90]	**< 0.001**
Kt/v	1.81 [1.62,2.04]	1.81 [1.62,2.04]	0.59
**TG** ≥**1.265**			**< 0.001**
No	70 (30%)	86 (65%)	
Yes	166 (70%)	46 (35%)	
4hD/Pcr	0.63 [0.58,0.69]	0.66 [0.60,0.69]	**0.002**
**Residual renal function**			0.66
No	116 (49%)	68 (52%)	
Yes	120 (51%)	64 (48%)	

Median (IQR); *n* (%), Wilcoxon rank sum test, Pearson's Chi-squared test. BMI, body mass index; DM, Diabetes Mellitus; ALB, albumin; TG, Triglycerides; HDL-C, High-density lipoprotein cholesterol; LDL-C, low-density. Lipoprotein cholesterol; CRP, C-reactive protein; kt/v, urea clearance index; 4hD/Pcr, the 4-h dialysate-to-plasma creatinine ratio; *p* < 0.05 are indicated in bold.

### 3.2 Factors Associated with Refractory hypoalbuminemia

2.1 Application of Box-Tidwell method test showed no linear relationship between plasma triglyceride level and logit transformed value of the dependent variable in PD patients. Therefore, the ROC curve was used to analyze the optimal cut-off value of TG to distinguish refractory hypoalbuminemia in PD patients, and the results suggested that the optimal cut-off value of TG was 1.265 mmol/L. The optimal cut-off value was further used to transform the continuous variable TG into a categorical variable. One hundred fifty-six cases (42.4%) of PD patients had a TG < 1.265 mmol/L. In the group of refractory hypoalbuminemia, the TG ≥ 1.265 mmol/L was the best cut-off value, while the TG ≥ 1.265 mmol/L in the PD group was the best cut-off value. 1.265 mmol/L was significantly reduced in the number of PD patients (65.2%), which was significantly different from the group without refractory hypoalbuminemia (*p* < 0.05) (Table 1).

2.2 Logistic regression analysis: diabetic comorbidity, application of compound α-keto acid treatment, gender, age, transmissible age, BMI, Kt/v, TG ≥1.265 mmol/L, HDL-C, residual renal function, 4hD/PCr, LDL-C, and CRP were included as independent variables in the multifactorial logistic regression analysis. The results suggested that diabetic comorbidity (OR: 4.20, 95%CI: 1.85~9.95), CRP (OR: 1.22, 95%CI: 1.12~1.35), and 4hD/Pcr (OR:4.37,95%CI: 2.41~8.42) were risk factors for refractory hypoalbuminemia in PD patients. Increased LDL-C (OR:0.338, 95%CI:0.188~0.607), HDL-C (OR: 0. 098, 95%CI: 0.024- 0.405), TG ≥1.265mmol/L (OR: 0.06, 95%CI: 0.02~0.15, and BMI (OR: 0.74, 95%CI: 0.67-0.82) were the factors of protective factors for refractory hypoalbuminemia (Figure 1A). Cardiovascular history was not significant on univariate analysis (*P* > 0.05), so excluded from the multivariate model.

**Figure 1:**
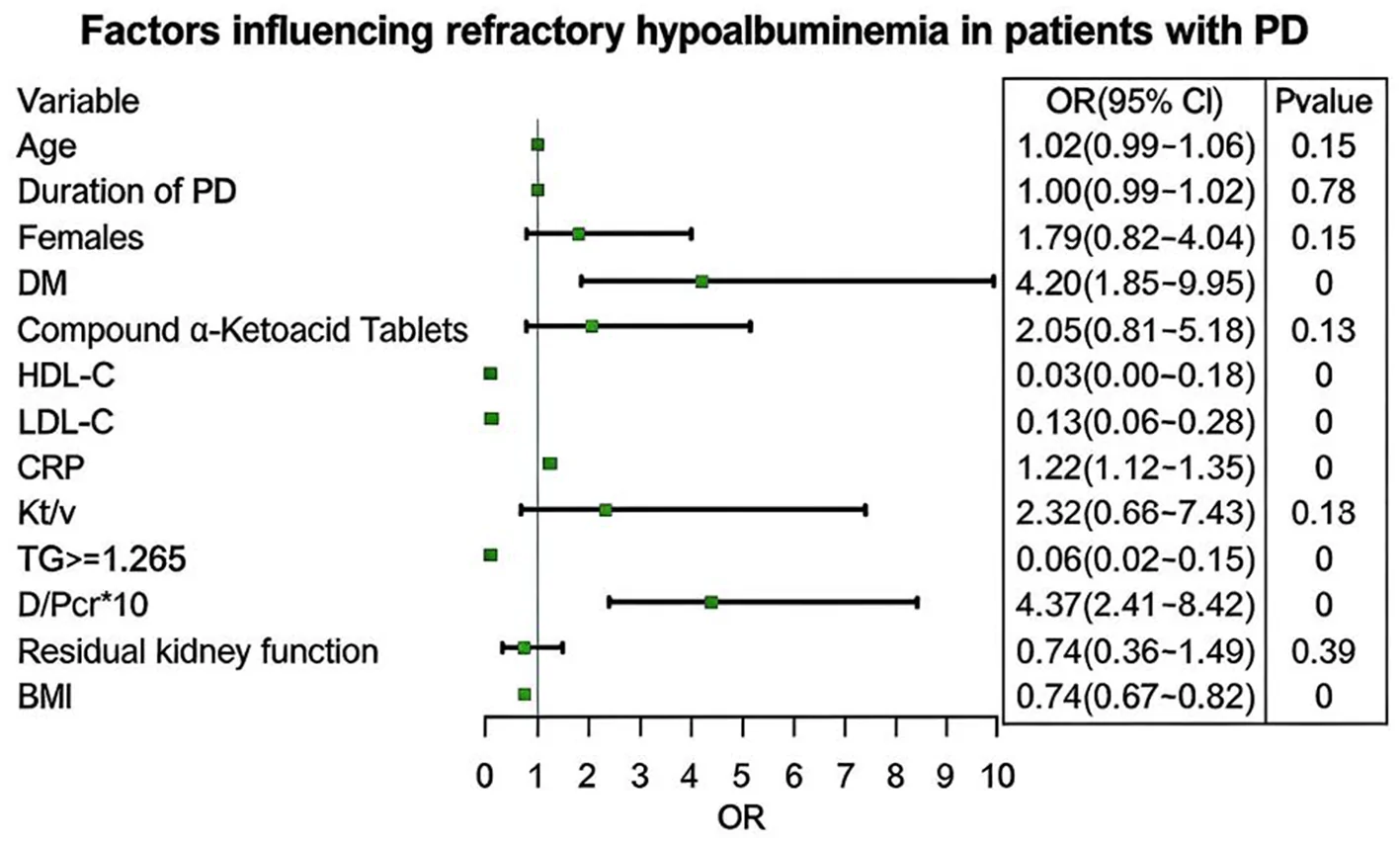
Factors influencing long-term hypoalbuminemia in patients with PD.

2.3 Propensity score matching: propensity score weighting was performed using patients with diabetes as the case group and non-diabetic patients as the control group, and the covariates of composite α-ketone acid treatment, gender, TG, BMI, HDL-C, CRP, Kt/v, age, residual renal function, 4hD/Pcr, dialysis time, and LDL-C. As shown in Figure 2A, the standardized percentage deviation of the covariates between the case and control groups after weighting correction was further reduced, and both were less than 20%, suggesting a good balance between the matched case and control groups. Table 2 shows the risk factors for refractory hypoalbuminemia in patients with diabetes mellitus still PD after weighted correction (*P* < 0.05). Similarly, patients taking oral compounded α-keto acids as the case group and patients not taking oral compounded α-keto acids as the control group were weighted with propensity score using comorbid diabetes mellitus, gender, TG, BMI, HDL-C, CRP, Kt/v, age, residual renal function, 4hD/Pcr, duration of dialysis and LDL-C as covariates. After weighting correction, there was a good balance between the case and control groups (Figure 2B). The analysis showed that the corrected application of compound α-keto acid treatment did not affect the occurrence of refractory hypoalbuminemia in PD patients (*p* = 0.375) (Table 2).

**Figure 2:**
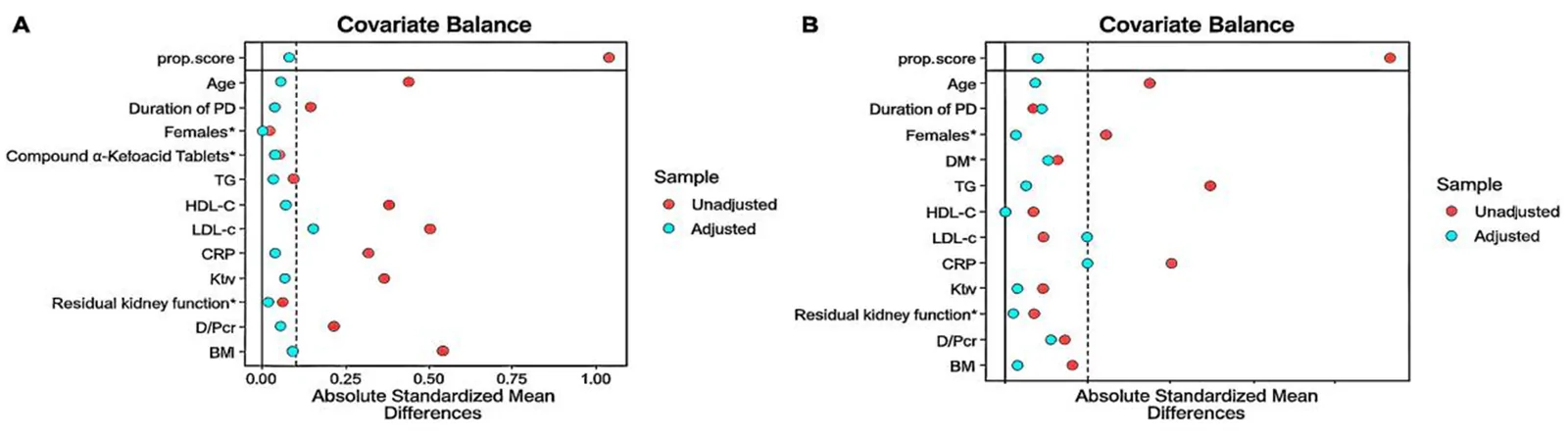
Balance test after propensity score weighting.

**Table 2:** Effect of diabetes and taking compounded α-keto acids on long-term hypoalbuminemia in PD patients after propensity score weighting.

Variable	Mode1	Mode2
Coefficients	0.6883	0.1364
*P*	**< 0.001**	0.375

Mode1, The effect of diabetes on refractory hypoalbuminemia after propensity score Weighting. Mode2, The effect of Compound α-Ketoacid Tablets on refractory hypoalbuminemia after propensity Score Weighting.

2.4 Among patients with PD combined with diabetes mellitus, patients with refractory hypoalbuminemia had higher glycated hemoglobin levels (*p* < 0.05, Figure 3A); 24-h quantitative urine protein levels were also analyzed in 59 cases with residual renal function, where 24-hour urine protein levels were higher in patients with refractory hypoalbuminemia (*p* < 0.05, Figure 3B).

**Figure 3:**
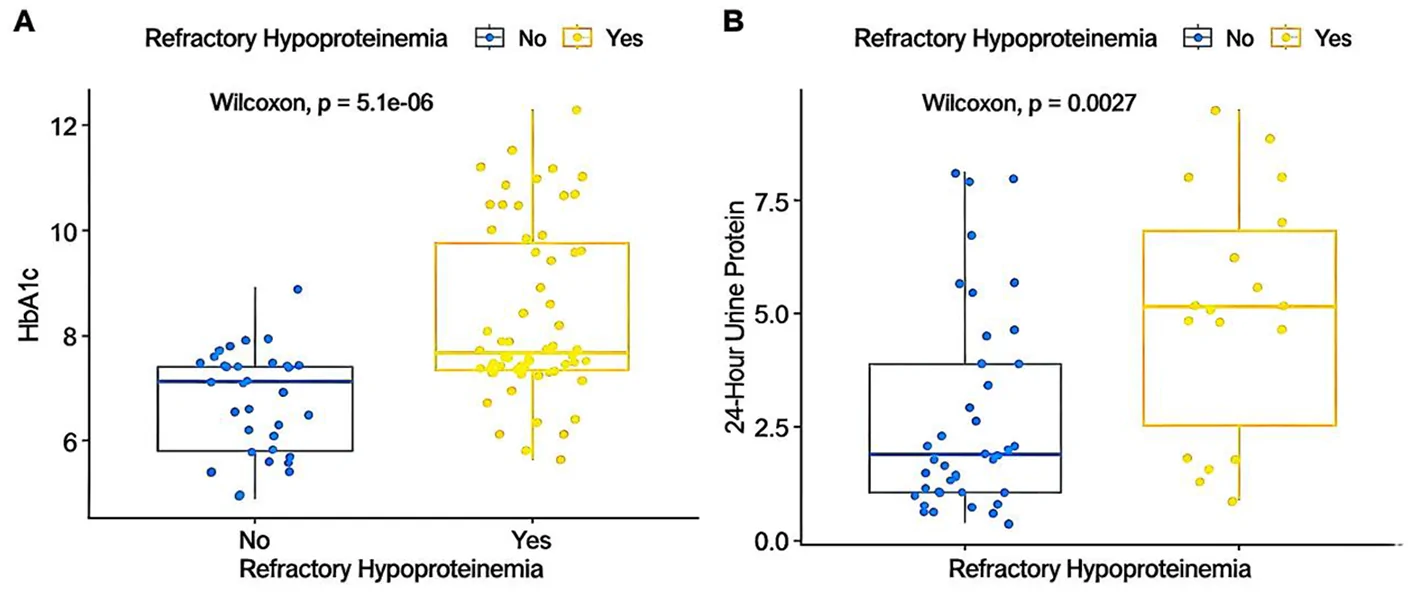
**(A)** Among diabetic PD patients, HbA1c levels were higher in patients with refractory hypoalbuminemia; **(B)** Among PD patients with residual renal function, 24-h urine protein levels were higher in patients with refractory hypoalbuminemia.

### 3.3 Construction and internal Validation of the Predictive Model

The final modeling cohort comprised 345 patients, of whom 111 (32.2%) had refractory hypoalbuminemia (Table 3).

**Table 3:** Baseline table of the modeling cohort.

**Variable**	No Refractory hypoalbuminemia *N* = 234	Refractory hypoalbuminemia *N* = 111	*p*-value
Age (years)	54 [44, 60]	55.00 [42, 60]	0.41
Females			0.45
Female	127 (54%)	65 (59%)	
Male	107 (46%)	46 (41%)	
BMI (kg/m2)	24.95 [18.90,26.80]	18.20 [17.00,21.00]	**< 0.001**
Duration of PD (months)	49.05 [32.40,69.90]	50.60 [36.80,70.80]	0.61
**Compound** α**-Ketoacid Tablets**			0.54
No	194 (83%)	89 (80%)	
Yes	40 (17%)	22 (20%)	
**DM**			**< 0.001**
No	203 (87%)	64 (58%)	
Yes	31 (13%)	47 (42%)	
ALB (g/L)	38.89 ± 2.48	31.51 ± 2.93	**< 0.001**
TG (mmol/L)	1.66 [1.28,2.40]	1.07 [0.79,1.56]	**< 0.001**
HDL-C (mmol/L)	1.13 [0.94,1.25]	0.90 [0.77,1.09]	**< 0.001**
LDL-C (mmol/L)	2.98 [2.44,3.51]	2.17 [2.01,2.54]	**< 0.001**
CRP (mg/L)	2.53 [1.83,4.59]	5.20 [2.75,11.20]	**< 0.001**
Kt/v	1.85 [1.66,2.09]	1.79 [1.62,2.05]	0.22
**TG** ≥**1.265**			**< 0.001**
No	57 (24%)	73 (66%)	
Yes	177 (76%)	38 (34%)	
4hD/Pcr	0.60 [0.58,0.65]	0.67 [0.63,0.69]	**< 0.001**
**Residual renal function**			0.87
No	118 (50%)	57 (51%)	
Yes	116 (50%)	54 (49%)	

Bold values indicate statistical significance (*P* < 0.05).

3.1 Screening of predictors: predictors were screened using the Lasso regression model. The distribution of coefficients for each predictor was plotted based on the log(λ) series (Figure 4A). Figure 4B represents the tuning of the parameters in the Lasso model, with the minimum criterion obtained by 10-fold cross-validation. The vertical dashed line on the left side indicates the model obtained using the minimum criterion, and the vertical dashed line on the right side indicates the model obtained with one standard error of the minimum criterion. Finally, we obtained seven best predictors, including diabetes mellitus comorbidity, TG ≥1.265 mmol/L, HDL-C, CRP, BMI, LDL-C, and 4hD/Pcr.

**Figure 4:**
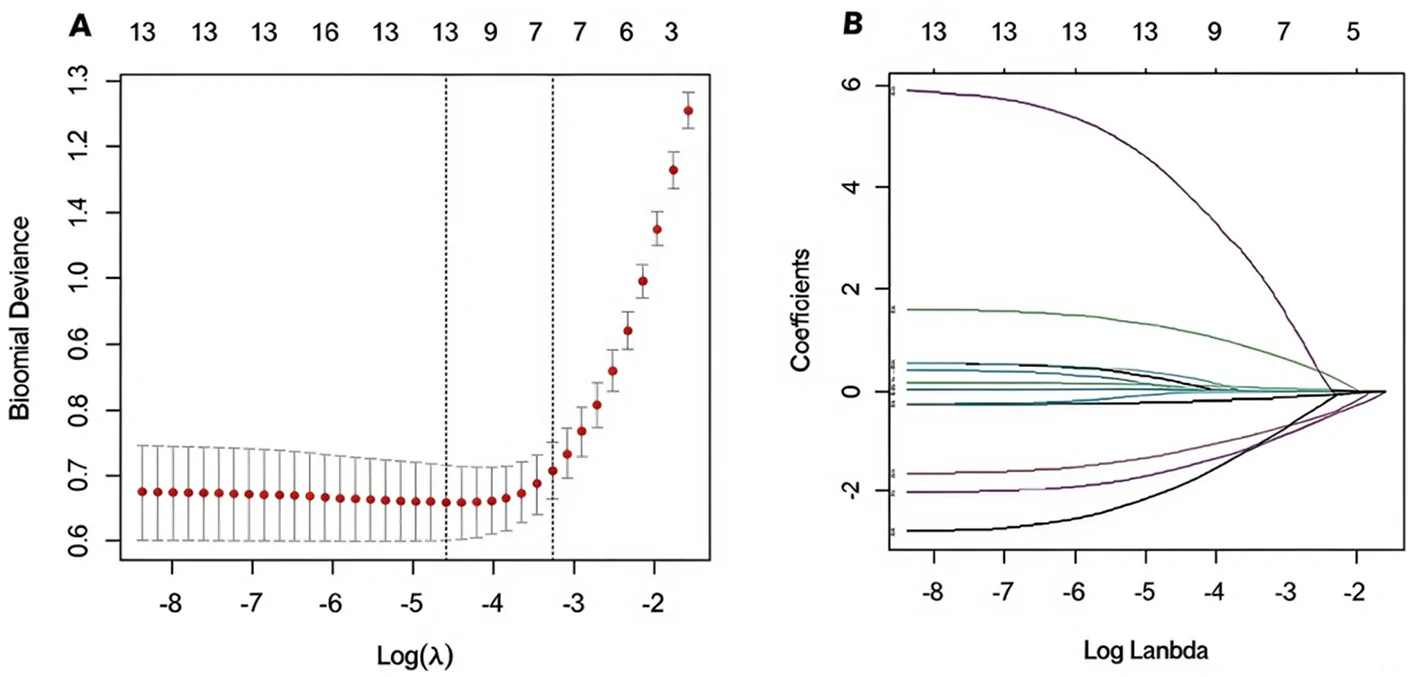
**(A)** Coefficient Distribution of Predictors; **(B)** Adjustment of parameters in Lasso regression models, the vertical dashed line on the left indicates the model obtained with the minimum criterion, and the vertical dashed line on the right shows the model obtained with one standard error of the minimum criterion.

3.2 Predictive model evaluation and internal validation: The best predictors mentioned above were included in the multifactor logistic regression analysis to construct the predictive model (Table 4); a nomogram was drawn based on the regression coefficients (Figure 5A). The predictive model had an area under the ROC curve of 0.939 in the modeling cohort (Figure 6A), a calibration curve close to a straight line with a slope of 1, and a brier score of 0.089 (< 0.25) (Figure 6B). Internal validation includes enhanced bootstrap (optimism 0.005; optimism-corrected AUC 0.934) and 10-fold stratified cross-validation (mean AUC 0.930; mean Brier score 0.092) (Table 5). We explicitly state that 10-fold cross-validation is an internal validation method and cannot replace independent external validation.

**Figure 5:**
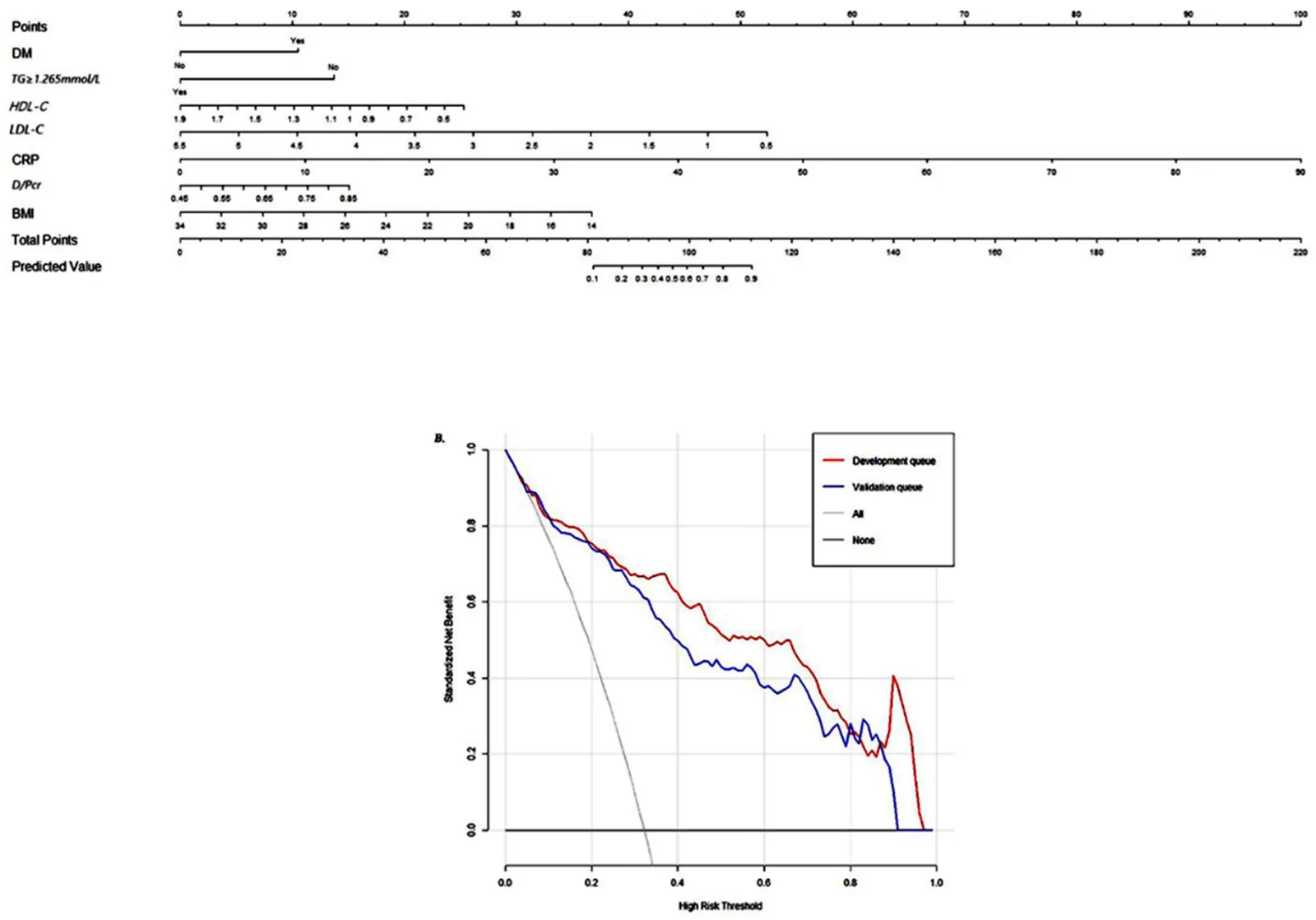
**(A)** The nomograph of predicting refractory hypoalbuminemia in PD patients; **(B)** Clinical decision curve analysis of prediction models in modeling cohorts and validation cohorts.

**Table 4:** Predictive model construction.

Characteristic	OR	95% CI^1^	*p*-value
DM	4.42	2.02 to 10.1	**< 0.001**
TG ≥ 1.265	0.14	0.06 to 0.31	**< 0.001**
HDL-C	0.09	0.02 to 0.46	**0.003**
LDL-c	0.23	0.12 to 0.41	**< 0.001**
CRP	1.17	1.09 to 1.27	**< 0.001**
4hD/Pcr	205	165 to 295	**0.030**
BMI	0.77	0.70 to 0.84	**< 0.001**

OR, Odds Ratio, CI, Confidence Interval. Bold values indicate statistical significance (*P* < 0.05).

**Figure 6:**
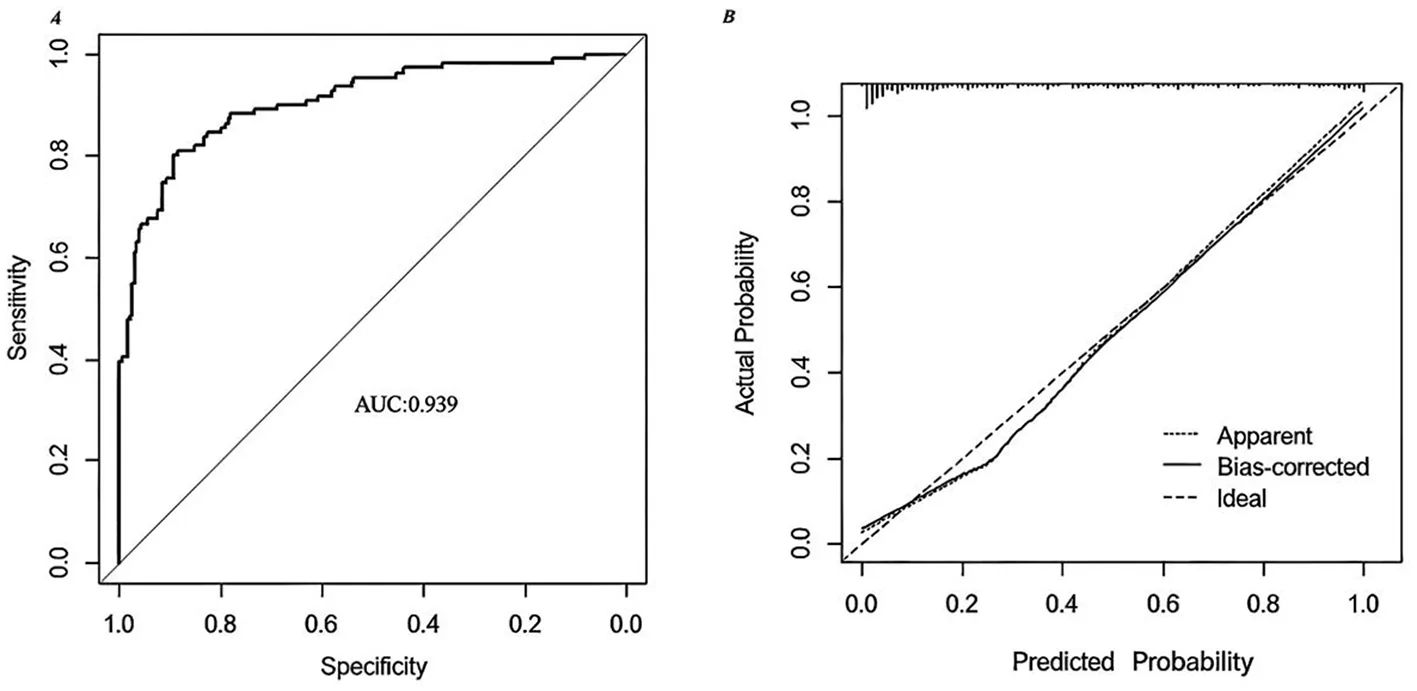
**(A)** The area under the ROC curve was 0.939; **(B)** Calibration curve of the predicted model in the modeling cohort and Brier score was 0.089.

**Table 5:** Internal validation of predictive models−10-fold cross-validation.

**Variable**	Modeling cohor	Bootstrap-corrected	10-fold cross-validation
AUC	0.939	0.934	0.930
Brier score	0.089	0.093	0.092
Optimism	–	0.005	–

The apparent AUC in the modeling cohort was 0.939, and the optimism-corrected AUC after bootstrap validation was 0.934. The mean AUC across the 10-fold cross-validation test sets was 0.930. The apparent Brier score was 0.089, the bootstrap-corrected Brier score was 0.093, and the mean cross-validated Brier score was 0.092. The AUC decreased by only 0.009 after cross-validation.

3.3 Testing the predictive model for clinical application: The performance of this predictive model for clinical application was analyzed using decision curves. Figure 5B suggests that there is an excellent net benefit in predicting the presence or absence of refractory hypoalbuminemia using a model based on the history of diabetes mellitus, BMI, blood CRP level, 4hD/Pcr, plasma HDL level, plasma LDL level, and blood triglyceride level, both in the modeling cohort and in the internal validation cohort, over a range of high-risk thresholds.

### 3.4 Peritoneal Protein Excretion Rate

A total of 95 PD patients with PD effluents were collected. The peritoneal protein excretion rate of PD patients with combined refractory hypoalbuminemia was significantly higher than that of PD patients without refractory hypoalbuminemia (*p* < 0.05, Figure 7A); PD patients with combined diabetes mellitus had a significantly higher peritoneal protein excretion rate than that of PD patients without diabetes mellitus (*p* < 0.05, Figure 7B); The peritoneal protein excretion rate was significantly positively correlated with 4hD/ Pcr levels. (*r* = 0.6, *p* < 0.05, Figure 7C)

**Figure 7:**
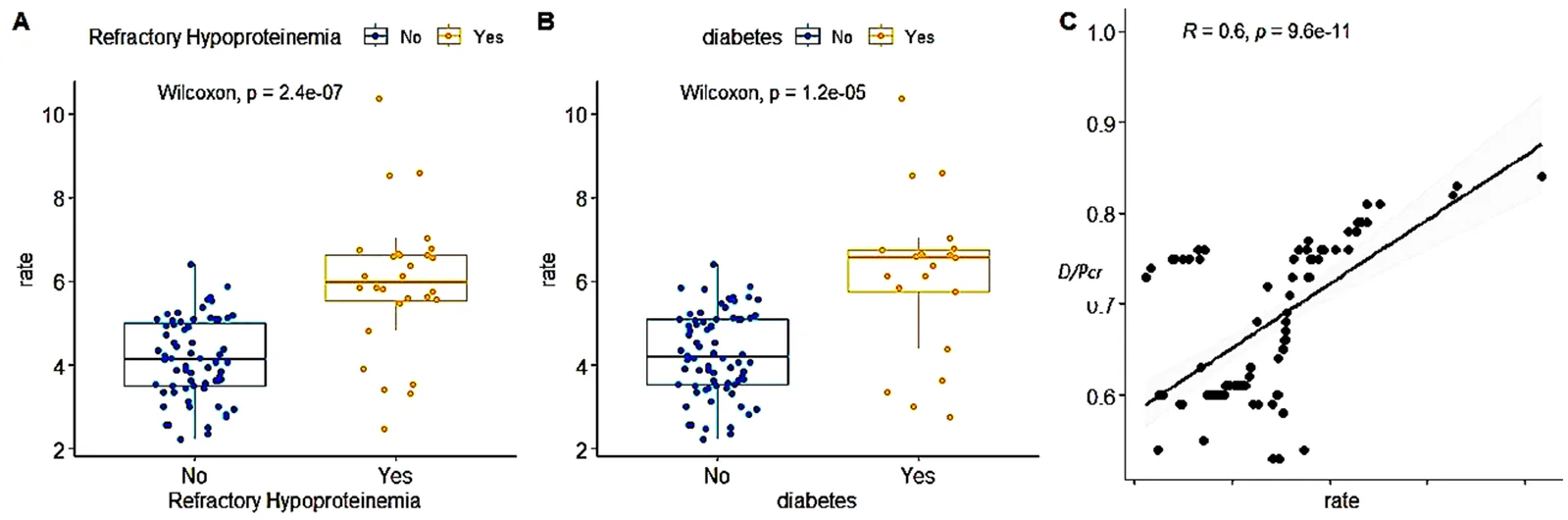
**(A)** Peritoneal protein excretion rate is higher in PD patients with refractory hypoalbuminaemia; **(B)** Peritoneal protein excretion rate is significantly increased in PD patients with diabetes. **(C)** Peritoneal protein excretion rate in PD patients positively correlates with their 4hD/Pcr levels, with a correlation coefficient 0.6.

## 4 Discussion

Regular monitoring of serum albumin levels is an essential indicator of the nutritional status of patients with PD. Changes in serum albumin levels during peritoneal dialysis (PD) are closely associated with patient survival. Studies have shown that a decrease in serum albumin can reduce survival in PD patients (11), This suggests that we should pay more attention to the correction of hypoalbuminemia in PD patients. In our clinical work, when we find PD patients with hypoalbuminemia, we will give nutritional interventions, such as oral whey protein powder, compound α-keto acid, and improve the dietary structure to increase protein intake in the diet. However, we found that in some patients with multiple nutritional interventions, their hypoalbuminemia still cannot be corrected, and it persists for an extended period. Our retrospective study found that diabetes mellitus, elevated C-reactive protein (CRP) levels, and a higher 4hD/Pcr were independent risk factors for refractory hypoalbuminemia in patients with PD, whereas higher BMI, TG, LDL-C, and HDL-C have a protective effect. The nomogram model constructed based on these factors demonstrates good discriminatory power and calibration, providing a convenient tool for the early clinical identification of high-risk patients.

Previous studies have confirmed that patients undergoing peritoneal dialysis lose protein through the dialysate at a rate of approximately 4-15g/d, which is one of the major causes of hypoalbuminemia in these patients (12, 13). The mechanism involves leakage of plasma proteins through the large peritoneal pores driven by peritoneal hydrostatic pressure during PD, leading to protein loss and increased risk of hypoalbuminemia (14). In addition, peritoneal protein loss is associated with inflammatory status, volume overload, residual renal function, and dialysis adequacy (15–17).

The 4-h dialysate-to-plasma creatinine ratio (4-h D/Pcr), measured during the peritoneal equilibration test, is the ratio of the dialysate creatinine concentration after a 4-h dwell to the plasma creatinine concentration at the same time point. Our study also found that the peritoneal protein excretion rate was positively correlated with the 4-h D/Pcr ratio in the peritoneal equilibrium test (*r* = 0.6, *P* < 0.05), suggesting that peritoneal protein loss may be increased in patients with high peritoneal transport status. Previous studies have similarly reported greater peritoneal protein losses and lower serum albumin levels in high transporters (18). We therefore hypothesize that the increased peritoneal protein loss rate in PD patients with refractory hypoalbuminemia may be related to their high peritoneal transport status. Furthermore, among patients with residual renal function, 24-h urinary protein excretion was significantly higher in those with refractory hypoalbuminemia. This finding highlights the importance of urinary protein losses in this population. Taken together, these findings suggest that excessive protein loss (via peritoneal and urinary routes) is an important cause of refractory hypoalbuminemia in PD patients.

Studies have found that protein losses through peritoneal dialysate are significantly greater in PD patients with diabetes than in those without diabetes (19). In this study, 47% of patients in the refractory hypoalbuminemia group had diabetes. Compared with PD patients without refractory hypoalbuminemia, those with refractory hypoalbuminemia had significantly higher rates of peritoneal protein excretion, suggesting that diabetes may be one of the risk factors for the development of refractory hypoalbuminemia in PD patients. Further analysis revealed that among PD patients with diabetes, those with refractory hypoalbuminemia had significantly elevated HbA1c levels. The mechanisms remain unclear but may be related to multiple factors, including microinflammation exacerbating insulin resistance, the conflict between nutritional support and glycemic control, and increased albumin permeability caused by diabetic microvascular complications (20, 21); Therefore, we therefore hypothesize that optimizing glycemic control may help prevent refractory hypoalbuminemia in PD patients with diabetes. However, the data collected thus far cannot directly explain why glycemic control was poorer in the refractory group, as we did not systematically collect information on medication adherence, dietary adherence, diabetes education, and socioeconomic status; thus, the impact of differences in adherence cannot be ruled out and requires further investigation in future studies.

PD patients often experience a state of low-grade inflammation due to various factors including reduced clearance of proinflammatory cytokines, increased oxidative stress, and acidosis (22, 23). Under chronic inflammatory conditions, the hepatic protein synthesis capacity is suppressed while catabolism is enhanced, making it difficult to maintain normal plasma albumin levels (10). Clinically, plasma C-reactive protein (CRP) levels are commonly used as a marke of systemic inflammation (24). In this study, we analyzed the relationship between plasma C-reactive protein (CRP) levels and refractory hypoalbuminemia in PD patients. The results revealed that as CRP levels rise, the risk of refractory hypoalbuminemia in PD patients increases significantly. Therefore, attention should be paid to the state of low-grade inflammation in patients with refractory hypoalbuminemia, and reducing inflammation may be an important component of managing refractory hypoalbuminemia in PD patients.

Compound α-ketoacid tablets are commonly used clinically to correct hypoalbuminemia and improve the nutritional status in patients receiving dialysis. However, the results of this study show that, after propensity score matching and adjustment for confounding factors such as inflammation in PD patients taking compound α-ketoacid tablets, oral administration of these tablets did not reduce the risk of refractory hypoalbuminemia in PD patients. Therefore, supplementation with compound α-keto acid tablets alone may not effectively correct refractory hypoalbuminemia in PD patients. Previous studies have similarly indicated that nutritional support alone is insufficient to correct hypoalbuminemia in critically ill patients (25). Therefore, the management of refractory hypoalbuminemia in patients undergoing PD should address not only protein intake and essential amino acid supplementation but also the contribution of low-grade inflammation. Inflammatory markers should be monitored, and patients should be assessed for persistent inflammation. Importantly, malnutrition and inflammation have a bidirectional relationship and may reinforce each other (26). Our study also showed that plasma cholesterol and triglyceride levels were significantly lower in patients with refractory hypoalbuminemia. Previous studies have reported a significant negative correlation between plasma cholesterol levels and plasma albumin levels, although the underlying mechanism remains unclear (27). Bologa et al. (28) found that plasma cholesterol levels were negatively correlated with levels of the inflammatory cytokine IL-6. Similarly, *in vitro* studies have shown that inflammatory cytokines inhibit hepatic lipoprotein and triglyceride synthesis and promote lipoprotein catabolism (29, 30). Furthermore, high-density lipoprotein (HDL) can inhibit endothelial activation by reducing the expression of adhesion molecules. Plasma HDL cholesterol levels may serve as a potential marker of underlying inflammatory processes (31). At the same time, this study also found that the risk of refractory hypoalbuminemia in PD patients increased significantly as BMI decreased. BMI is commonly used as an indicator of nutritional status, and a low BMI generally suggests malnutrition. Therefore, we speculate that the lower plasma cholesterol and triglyceride levels in PD patients with refractory hypoalbuminemia may be the result of the combined effects of a state of low-grade inflammation and malnutrition.

Persistent volume overload has also been reported to be significantly associated with lower serum albumin levels and is considered an important contributor to hypoalbuminemia in this population (8, 32, 33). Volume overload may directly lead to hypoalbuminemia through mechanisms such as volume expansion, increased capillary permeability, and abnormal fluid distribution (34, 35). It may also aggravate hypoalbuminemia by increasing transmembrane albumin loss. Kwan et al. found that in PD patients assessed by bioelectrical impedance analysis (BIA), fluid overload was associated with higher peritoneal permeability (36). Thus, the elevated 4-hD/Pcr observed in patients with refractory hypoalbuminemia may not only reflect intrinsic peritoneal transport characteristics but may also be partly attributable to fluid overload. This suggests that fluid status should be evaluated concurrently when assessing hypoalbuminemia in PD patients in clinical practice. However, our center did not systematically evaluate patients'fluid status using objective methods such as bioimpedance analysis (BIA). The fluid-related variables we collected were limited to clinical assessment indicators, including changes in body weight, the presence of edema, blood pressure, ultrafiltration rate, and a history of heart failure. Notably, all patients judged to have substantial volume overload based on these clinical assessments were excluded from the study. Consequently, the potential effect of volume overload on hypoalbuminemia was likely attenuated to some extent, although this issue warrants further investigation.

In summary, refractory hypoalbuminemia in PD patients is influenced by multiple factors. Hypoalbuminemia is associated with a significantly increased risk of cardiovascular events, poorer prognosis, and a greater economic burden on both patients and society. To facilitate the early identification of high-risk patients and timely intervention, we developed a clinical prediction model for refractory hypoalbuminemia in PD patients. The ultimate aim was to help reduce its incidence and improve patient outcomes. The model incorporated comorbid diabetes mellitus, C-reactive protein (CRP), triglycerides (TG) ≥1.265 mmol/L, body mass index (BMI), low-density lipoprotein cholesterol (LDL-C), the 4-h dialysate-to-plasma creatinine ratio (4-h D/PCr) measured during the peritoneal equilibration test, and high-density lipoprotein cholesterol (HDL-C).A nomogram was constructed to provide a visual assessment of individual risk. All variables included in the nomogram are routinely assessed and readily available in clinical practice. Its graphical format also eliminates the need for complex calculations. To use the nomogram, clinicians first assign points to each predictor according to the patient's values and then sum these points to obtain a total score. The total score is subsequently mapped to the corresponding predicted probability to estimate the patient's risk of refractory hypoalbuminemia. Decision curve analysis suggested that the model had potential clinical utility. This model may facilitate the early identification of patients at risk of refractory hypoalbuminemia and support timely, individualized interventions targeting the underlying contributing factors. It may therefore serve as a convenient and useful tool for clinical management.

## 5 Study limitations

This study has several limitations. First, as a single-center retrospective study, all patients were recruited from the same medical center. The relatively homogeneous study population in terms of geographic region, ethnicity, socioeconomic background, and treatment protocols may limit the generalizability of our findings to other settings. Second, no independent external validation was conducted. The temporal cohort overlapped with the modeling cohort at the patient level, so it could not function as an independent external validation cohort. Third, although this study identified associations between the examined factors and refractory hypoalbuminemia, it could not establish causal relationships. Malnutrition–inflammation–atherosclerosis (MIA) syndrome is common in patients undergoing PD. Although we collected data on cardiovascular disease history, univariate analysis did not show a significant association between this factor and refractory hypoalbuminemia, possibly due to the limited sample size or incomplete medical record documentation. Furthermore, objective measures of atherosclerosis, such as carotid intima–media thickness, were not included, which restricts the model's ability to capture the full spectrum of MIA syndrome. Therefore, the prediction model requires external validation in larger, multicenter, prospective studies with non-overlapping patient populations to fully evaluate its generalizability and clinical applicability.

## 6 Conclusion

This study demonstrated that diabetes mellitus, LDL cholesterol, c-reactive protein, TG≥1.265 mmol/L, BMI, 4hD/Pcr, and HDL cholesterol were influential factors for refractory hypoalbuminemia in patients with PD. Higher rate of peritoneal protein excretion in PD patients with refractory hypoalbuminemia. The clinical prediction model constructed based on these factors can help identify refractory hypoalbuminemia of high risk and with high external applicability. In the future, it is necessary to expand further on the influencing factors of refractory hypoalbuminemia in PD patients to apply this prediction model in clinical practice and to guide individualized treatment.

## Data Availability

The raw data supporting the conclusions of this article will be made available by the authors, without undue reservation.

## References

[1] Bassuner J Kowalczyk B. Why peritoneal dialysis is underutilized in the United States: a review of inequities. Semin Intervent Radiol. (2022) 39:47–50. doi: 10.1055/s-0041-1741080 35210732 PMC8856784

[2] Zhao Y. Comparison of the effect of hemodialysis and peritoneal dialysis in the treatment of end-stage renal disease. Pak J Med Sci. (2023) 39:1562–67. doi: 10.12669/pjms.39.6.8056 37936738 PMC10626077

[3] Wang X Zhang M Sun N Chang W. Effects and clinical value of peritoneal dialysis on water and water balance, adverse reactions, quality of life, and clinical prognosis in patients with decompensated chronic nephropathy: a systematic review and meta-analysis. Comput Math Methods Med. (2022) 2022:9469134. doi: 10.1155/2022/9469134 35898489 PMC9313948

[4] Li PK Chow KM Van de Luijtgaarden MW Johnson DW Jager KJ Mehrotra R . Changes in the worldwide epidemiology of peritoneal dialysis. Nat Rev Nephrol. (2017) 13:90–103. doi: 10.1038/nrneph.2016.181 28029154

[5] Changsirikulchai S Sriprach S Thokanit NS Janma J Chuengsaman P Sirivongs D. Survival analysis and associated factors in Thai patients on peritoneal dialysis under the PD-first policy. Perit Dial Int. (2018) 38:172–78. doi: 10.3747/pdi.2017.00127 29437140

[6] Foley RN Parfrey PS Harnett JD Kent GM Murray DC Barre PE. Hypoalbuminemia, cardiac morbidity, and mortality in end-stage renal disease. J Am Soc Nephrol. (1996) 7:728–36. doi: 10.1681/ASN.V75728 8738808

[7] Tsai HL Yang LY Chin TW Wang HH Liu CS Wei CF . Outcome and risk factors for mortality in pediatric peritoneal dialysis. Perit Dial Int. (2010) 30:233–39. doi: 10.3747/pdi.2008.00278 20150582

[8] Banno T Shima H Kawahara K Okada K Minakuchi J. Risk factors for peritoneal dialysis withdrawal due to peritoneal dialysis-related peritonitis. Nephrol Ther. (2021) 17:108–13. doi: 10.1016/j.nephro.2020.10.007 33495136

[9] Song P Yang D Li J Zhuo N Fu X Zhang L Zhang H . Dynamic serum albumin and outcome of peritoneal dialysis patients: a retrospective study in China. Front Med (Lausanne). (2022) 9:917603. doi: 10.3389/fmed.2022.917603 35983095 PMC9381003

[10] Jin L Wang X Ma Y Zheng J Lu W Xie L . Serum albumin at 1 year after peritoneal dialysis predicts long-term outcomes on continuous ambulatory peritoneal dialysis. Ren Fail. (2022) 44:252–57. doi: 10.1080/0886022X.2022.2033264 35166186 PMC8856101

[11] Chiu PF Tsai CC Wu CL Wu CL Yang TY Liou HH . Trajectories of serum albumin predict survival of peritoneal dialysis patients: a 15-year follow-up study. Medicine (Baltimore). (2016) 95:e3202. doi: 10.1097/MD.0000000000003202 27015223 PMC4998418

[12] Rippe B Öberg CM. Albumin turnover in peritoneal and hemodialysis. Semin Dial. (2016) 29:458–62. doi: 10.1111/sdi.12534 27525385

[13] Kagan A Bar-Khayim Y. Role of peritoneal loss of albumin in the hypoalbuminemia of continuous ambulatory peritoneal dialysis patients: relationship to peritoneal transport of solutes. Nephron. (1995) 71:314–20. doi: 10.1159/000188738 8569981

[14] Malho Guedes A Calças Marques R Domingos AT Laranjo C Silva AP Rodrigues A . Peritoneal protein loss with time in peritoneal dialysis. Semin Dial. (2024) 37:242–48. doi: 10.1111/sdi.13194 38420712

[15] Malho Guedes A Calças Marques R Ribeiro B Fernandes MT Faísca M Silva AP . Peritoneal protein loss, inflammation, and nutrition: refuting myths. Front Med (Lausanne). (2022) 9:884061. doi: 10.3389/fmed.2022.884061 35692552 PMC9178188

[16] Yu Z Lambie M Chess J Williams A Do JY Topley N . Peritoneal protein clearance is a function of local inflammation and membrane area whereas systemic inflammation and comorbidity predict survival of incident peritoneal dialysis patients. Front Physiol. (2019) 10:105. doi: 10.3389/fphys.2019.00105 30833904 PMC6387967

[17] Qin A Liu X Yin X Zhou H Tang Y Qin W. Normalized protein catabolic rate is a superior nutritional marker associated with dialysis adequacy in continuous ambulatory peritoneal dialysis patients. Front Med (Lausanne). (2021) 7:603725. doi: 10.3389/fmed.2020.603725 33511142 PMC7835658

[18] Guan JC Bian W Zhang XH Shou ZF Chen JH. Influence of peritoneal transport characteristics on nutritional status and clinical outcome in Chinese diabetic nephropathy patients on peritoneal dialysis. Chin Med J (Engl). (2015) 128:859–64. doi: 10.4103/0366-6999.154275 25836603 PMC4833999

[19] Nakamoto H Imai H Kawanishi H Nakamoto M Minakuchi J Kumon S . Effect of diabetes on peritoneal function assessed by personal dialysis capacity test in patients undergoing CAPD. Am J Kidney Dis. (2002) 40:1045–54. doi: 10.1053/ajkd.2002.36343 12407651

[20] Fujihara R Chiba Y Nakagawa T Nishi N Murakami R Matsumoto K . Albumin microvascular leakage in brains with diabetes mellitus. Microsc Res Tech. (2016) 79:833–37. doi: 10.1002/jemt.22708 27333535

[21] Khalil H. Diabetes microvascular complications—a clinical update. Diabetes Metab Syndr. (2017) 11:S133–39. doi: 10.1016/j.dsx.2016.12.022 27993541

[22] Akchurin OM Kaskel F. Update on inflammation in chronic kidney disease. Blood Purif. (2015) 39:84–92. doi: 10.1159/000368940 25662331

[23] Bonanni A Mannucci I Verzola D Sofia A Saffioti S Gianetta E Garibotto G. Protein-energy wasting and mortality in chronic kidney disease. Int J Environ Res Public Health. (2011) 8:1631–54. doi: 10.3390/ijerph8051631 21655142 PMC3108132

[24] Moutachakkir M Lamrani Hanchi A Baraou A Boukhira A Chellak S. Immunoanalytical characteristics of C-reactive protein and high sensitivity C-reactive protein. Ann Biol Clin (Paris). (2017) 75:225–29. doi: 10.1684/abc.2017.1232 28377336

[25] Soeters PB Wolfe RR Shenkin A. Hypoalbuminemia: pathogenesis and clinical significance. JPEN J Parenter Enteral Nutr. (2019) 43:181–93. doi: 10.1002/jpen.1451 30288759 PMC7379941

[26] Kalantar-Zadeh K Ikizler TA Block G Avram MM Kopple JD. Malnutrition-inflammation complex syndrome in dialysis patients: causes and consequences. Am J Kidney Dis. (2003) 42:864–81. doi: 10.1016/j.ajkd.2003.07.016 14582032

[27] Chiarla C Giovannini I Giuliante F Zadak Z Vellone M Ardito F . Severe hypocholesterolemia in surgical patients, sepsis, and critical illness. J Crit Care. (2010) 25:361.e7–361.e12. doi: 10.1016/j.jcrc.2009.08.006 19828282

[28] Bologa RM Levine DM Parker TS Cheigh JS Serur D Stenzel KH . Interleukin-6 predicts hypoalbuminemia, hypocholesterolemia, and mortality in hemodialysis patients. Am J Kidney Dis. (1998) 32:107–14. doi: 10.1053/ajkd.1998.v32.pm9669431 9669431

[29] Song H Saito K Fujigaki S Noma A Ishiguro H Nagatsu T . IL-1β and TNF-α suppress apolipoprotein (apo) E secretion and apo A-I expression in HepG2 cells. Cytokine. (2002) 17:246–53. 10.1006/cyto.1997.0291 9617572

[30] Ettinger WH Varma VK Sorci-Thomas M Parks JS Sigmon RC Smith TK . Cytokines decrease apolipoprotein accumulation in medium from Hep G2 cells. Arterioscler Thromb. (1994) 14:8–13. doi: 10.1161/01.ATV.14.1.8 8274481

[31] Golucci APBS Marson FAL Ribeiro AF Nogueira RJN. Lipid profile associated with the systemic inflammatory response syndrome and sepsis in critically ill patients. Nutrition. (2018) 55-56:7–14. doi: 10.1016/j.nut.2018.04.007 29960160

[32] Ng JKC Kwan BCH Chow KM Pang WF Cheng PMS Leung CB . Asymptomatic fluid overload predicts survival and cardiovascular event in incident Chinese peritoneal dialysis patients. PLoS One. (2018) 13:e0202203. doi: 10.1371/journal.pone.0202203 30102739 PMC6089451

[33] Ng JKC Kwan BCH Chan GCK Chow KM Pang WF Cheng PMS . Predictors and prognostic significance of persistent fluid overload: a longitudinal study in Chinese peritoneal dialysis patients. Perit Dial Int. (2023) 43:252–62. doi: 10.1177/08968608221110491 35787209

[34] van Gelder MK Abrahams AC Joles JA Kaysen GA Gerritsen KGF. Albumin handling in different hemodialysis modalities. Nephrol Dial Transplant. (2018) 33:906–13. doi: 10.1093/ndt/gfx191 29106652

[35] Kooman JP van der Sande FM. Body fluids in end-stage renal disease: statics and dynamics. Blood Purif. (2019) 47:223–29. doi: 10.1159/000494583 30517920 PMC6492508

[36] Kwan BC Szeto CC Chow KM Law MC Cheng MS Leung CB . Bioimpedance spectroscopy for the detection of fluid overload in Chinese peritoneal dialysis patients. Perit Dial Int. (2014) 34:409–16. doi: 10.3747/pdi.2013.00066 24385329 PMC4079487

